# Characterization and Cytotoxicity Comparison of Silver- and Silica-Based Nanostructures

**DOI:** 10.3390/ma14174987

**Published:** 2021-08-31

**Authors:** Elżbieta Adamska, Karolina Niska, Anna Wcisło, Beata Grobelna

**Affiliations:** 1 Faculty of Chemistry, University of Gdansk, Wita Stwosza 63, 80-308 Gdańsk, Poland; elzbieta.adamska@ug.edu.pl (E.A.); anna.wcislo@ug.edu.pl (A.W.); 2 Department of Medical Chemistry, Faculty of Medicine, Medical University of Gdańsk, Debinki St., 80-210 Gdańsk, Poland; niska.karolina@gumed.edu.pl

**Keywords:** silver nanoparticles, silica coatings, core-shell structures, nanostructures cytotoxicity

## Abstract

Core-shell structures are the most common type of composite material nanostructures due to their multifunctional properties. Silver nanoparticles show broad antimicrobial activity, but the safety of their utilization still remains an issue to tackle. In many applications, the silver core is coated with inorganic shell to reduce the metal toxicity. This article presents the synthesis of various materials based on silver and silica nanoparticles, including SiO_2_@Ag, Ag@SiO_2_, and sandwich nanostructures—Ag@SiO_2_@Ag—and the morphology of these nanomaterials based on transmission electron microscopy (TEM), UV-Vis spectroscopy, and FT-IR spectroscopy. Moreover, we conducted the angle measurements due to the strong relationship between the level of surface wettability and cell adhesion efficiency. The main aim of the study was to determine the cytotoxicity of the obtained materials against two types of human skin cells—keratinocytes (HaCaT) and fibroblasts (HDF). We found that among all the obtained structures, SiO_2_@Ag and Ag@SiO_2_ showed the lowest cell toxicity and very high half-maximal inhibitory concentration. Moreover, the measurements of the contact angle showed that Ag@SiO_2_ nanostructures were different from other materials due to their superhydrophilic nature. The novel approach presented here shows the promise of implementing core-shell type nanomaterials in skin-applied cosmetic or medical products.

## 1. Introduction

In recent years, nanosilver (Ag NPs) has often been used in everyday products, including dressings [1,2,3], cleaning agents [4], cosmetics [5,6], and controlled drug delivery [7] due to its enhanced antibacterial or antifungal properties compared with macro-scale silver. Moreover, silver nanoparticles have unique properties, such as optical properties concerning the surface plasmon phenomenon [8]. Despite the many advantages of Ag NPs, there are more scientific publications indicating its toxic properties, which are influenced by many factors, including those that are most important, such as size [6], shape [9], or the type of nanoparticle synthesis [10].

Due to the absolute safety requirements of the marketed products, materials that combine the advantages of nanoparticles and low toxicity are highly required. The solution to this problem is the modification of nanoparticles with inorganic compounds, e.g., silica (SiO_2_) [11]. In addition, silica-based nanoparticles exhibit strong biological activities, for example, promotion of osteoblasts, adhesion, proliferation, and stimulation of the osteogenic differentiation in vivo [12,13,14].

The type of obtained nanostructures that are composed of two materials are called core-shell nanostructures and can be divided into various categories according to their physicochemical properties—for example, core-shell nanoparticles, core-shell magnetics, core-shell silica nanoparticles, or core-shell polymer nanoparticles [15]. Taking into account recent scientific reports, the interest of scientists is focused primarily on silica-covered metal structures, the most frequently studied of which are Ag@SiO_2_ [8,16,17], Au@SiO_2_ [18,19,20], or TiO_2_@SiO_2_ [21,22,23]. It is worth mentioning that core-shell nanomaterials are not only less cytotoxic but also show high dispersibility, biocompatibility, and improved thermal and chemical stability [7]. Importantly, scientific reports show that silica-coated metals do not lose their unique properties, such as the antimicrobial properties of Ag NPs in Ag@SiO_2_ structures [24,25]. Moreover, the importance of the hydrophilicity of core-shell nanostructures in biological applications is also well known [26].

The complex morphology of nanostructures can be examined using various methods. The standard techniques are electron microscopy—including scanning (SEM) or transmission (TEM), Fourier-transform infrared spectroscopy (FT-IR), or thermogravimetric analysis (TG). In addition, all the methods are under constant development, which allows accurate characterization of the nanoparticle surfaces, including the determination of active groups number [27].

One of the nanoparticles’ description parameters is their potential toxicity, including the determination of an exposure route. There are several routes of human exposure to nanoparticles [28,29,30], including pulmonary [31,32,33], oral [34,35,36,37], and transdermal [6,38,39,40]. One of the most susceptible routes of nanoparticles action is the transdermal route. This is not only due to the large number of cosmetic and medical products utilizing nanostructures but also because of the small size of nanoparticles. We currently know that penetration of nanoparticles by the percutaneous route can occur in two ways: through the hair follicles and through the spaces between corneocytes [5]. Since the first barrier on the skin that nanoparticles come into contact with is the stratum corneum, corneocytes are the most susceptible to them. Moreover, some studies indicate that nanoparticles can penetrate deeper, i.e., into the vicinity of fibroblasts, which in turn enables the penetration of the blood vessels and distribution in the whole organism [6].

However, due to the relative novelty of core-shell structures and despite the insight in surface and deep skin cells cytotoxicity, we lack studies on the safety of nanomolecules. In the present work, we synthesized nanoparticles of silver, silica, and core-shell structures with various silver and silica configurations—SiO_2_@Ag, Ag@SiO_2_, and Ag@SiO_2_@Ag. Due to differences in potential penetration depth, the cytotoxicity of these structures was determined on two human skin cell lines: keratinocytes (HaCaT) and fibroblasts (HDF).

## 2. Materials and Methods

### 2.1. Materials and Reagents

All chemicals were of analytical grade and were used without further purification. Acetone, ammonia solutions (25%), cetyltrimethylammonium bromide (CTAB), ethanol, hydrazine hydrate (N_2_H_4_·H_2_O), poly(vinylpolypyrrolidone) (PVP), silver nitrate, (AgNO_3_, 99.99%), sodium borohydride (NaBH_4_), tetraethyl orthosilicate (TEOS), and trisodium citrate (Na-cit) were purchased from Sigma-Aldrich (Poznan, Poland). All the samples were prepared using deionized water produced using a Hydrolab system installed in our laboratory.

The human dermal fibroblasts (HDF) were purchased from Cell Applications, Inc. (San Diego, CA, USA), and spontaneously immortalized human keratinocytes from histologically normal skin (HaCaT) were a kind gift from Professor Michal Zmijewski (Medical University of Gdansk, Poland). HDF and HaCaT cells were cultured in Dulbecco’s modified Eagle’s medium (DMEM) with high glucose, supplemented with 10% fetal bovine serum (FBS), 6 μg/mL penicillin-G, and 10 μg/mL streptomycin under controlled conditions of 37 °C in a humidified atmosphere containing 5% CO_2_. When 80–90% confluency was obtained, cells were enzymatically detached with trypsin-EDTA and transferred into new T-75 cm^2^ tissue culture flasks.

### 2.2. Synthesis

#### 2.2.1. Synthesis of Silver Nanoparticles (Ag NPs)

There are many methods available for silver nanoparticles synthesis. Most of them are based on a reduction in silver nitrate, with sodium borohydride as a reducing agent. In our case, silver nanoparticles were prepared according to the method described previously by Szczepańska et al. [6]. First, we prepared 60 mL of 0.002 M sodium borohydride and cooled it in an ice bath for half an hour. Then, 20 mL of 0.001 M silver nitrate was slowly added to the solution with stirring. The synthesis was continued until the color of the solution changed to yellow.

#### 2.2.2. Synthesis of Silica Nanoparticles (SiO_2_ NPs)

In order to obtain SiO_2_ NPs, the method presented by Sakthisabarimoorthi et al. was modified [41]. To 80 mL of ethanol was added 2.5 mL of ammonia water and 5 mL of water with vigorous stirring. The prepared solution was then combined with 5 mL of TEOS. Reactions were carried out for 24 h at room temperature with constant stirring. The solution was separated by centrifugation at 6000 rpm for 20 min and washed several times with ethanol.

#### 2.2.3. Synthesis of SiO_2_@Ag

SiO_2_@Ag was obtained according to the method described by Sakthisabarimoorthi et al. [41]. The dried SiO_2_ NPs (0.05 g) were dissolved in 20 mL of ethanol followed by the addition of 15 mL of 0.02 M silver nitrate with 1 mL of ammonia solution. After 20 min, 10 mL of PVP was added and allowed to mix. Then, 5 mL of 0.02 M NaBH_4_ was injected into the mixture, which was stirred for 0.5 h at room temperature. SiO_2_@Ag was collected by centrifugation at 6000 rpm for 20 min and washed with ethanol and deionized water.

#### 2.2.4. Synthesis of Ag@SiO_2_

Ag@SiO_2_ nanostructures were prepared according to the method described by Synak et al. [8]. Initially, 40 mL of Ag NPs, 20 mL of ethanol, and 0.25 mL of sodium citrate (0.3 M) were mixed. To adjust the pH to about 9, a small amount of ammonia water was added. Subsequently, the solution was mixed with 10 mL of TEOS. The Ag@SiO_2_ structures were collected by centrifugation and washed with a mixture of water and ethanol.

#### 2.2.5. Synthesis of Ag@SiO_2_@Ag

Ag@SiO_2_@Ag structures were obtained based on the modified method of Zhao et al. [16]. Amounts of 1.5 mL of hydrazine solution and 0.9 mmol of CTAB were added to 250 mL of distilled water, with vigorous stirring for several minutes at room temperature. A silver nitrate solution (1.5 mmol, 10 mL) was then prepared, which was added dropwise to the solution obtained above. Then, 50 mL of ethanol, 2.5 mL of aqueous ammonia solution, and 0.5 mL of TEOS were added successively with stirring. The reaction was carried out for 8 h. After this time, the resulting structures were separated by centrifugation and washed several times with ethanol and water.

### 2.3. Methods

Transmission electron microscopy (TEM) images were made using a Tecnai G2 Spirit BioTWIN by FEI. UV-Vis Spectroscopy spectra were obtained using a Perkin-Elmer Lambda 18 (wavelength accuracy ± 0.1 nm, resolution 0.1 nm, measurement range 300–700 nm). FT-IR spectroscopy analysis was carried out using a Bruker IFS66 spectrometer.

The thermal analysis of the nanostructures was conducted using Jupiter STA 449 F3 thermogravimetry connected to a QMS 403 C quadrupole mass spectrometer (NETZSCH, Germany). The measurements were carried out in an inert gas (nitrogen) atmosphere, from room temperature to 1000 °C, with a heating rate of 10 °C/min.

The contact angle measurements were made using a Kruss (Hamburg, Germany) DSA100 goniometer. Prior to measurement, a suspension of nanoparticles in ethanol (dispersed by ultrasound) was applied to a glass plate and allowed to evaporate at room temperature. The thus formed nanoparticle surface was subjected to further tests. A water drop of 2 µL volume was applied with a syringe to the surface made of the tested nanoparticles. The contact angle on both sides of the droplet was measured by a CCD camera, and after digital image analysis, the average contact angle was determined using Young–Laplace method. Each measurement was repeated 20 times [42,43,44,45].

Before testing for cytotoxicity, all structures were subjected to multiple purification of organic impurities, primarily through multiple washing of nanoparticles and drying. Cell viability was determined using MTT (3-(4,5-dimethylthiazol-2-yl)-2,5-diphenyltetrazolium bromide) assay. The cells were seeded into 96-well microplates with 12–14 × 10^3^/100 µL cells per well. The following day, fibroblast and keratinocyte cells were exposed to various concentrations of silver and silica-based nanostructures prepared ex tempore in serum-free cell culture medium and incubated for 24 h. Then, tetrazolium dye (MTT) was added to each well. After 2 h, medium was removed and obtained formazan crystals were dissolved in dimethylsulfoxide (DMSO). The absorbance was recorded at 492 nm. The data are presented as a percentage of the control values (untreated cells), which was set as 100%.

The experimental data are presented as the mean ± SD for at least triplicate determination of three independent experiments. The obtained results were analyzed using commercially available software (GraphPad Prism 5) based on one-way ANOVA and Tukey’s post hoc test. *p* values < 0.05 were considered statistically significant. The IC_50_ was determined by nonlinear regression analysis: log (inhibitor) vs. normalized response.

## 3. Results and Discussion

The design of new classes of materials, especially for medicine or cosmetology, constantly challenges scientists working in the field of materials science. Silver and gold nanoparticles have been studied for decades because of their optical [46], magnetic [47], and antimicrobial properties [6]. Despite the numerous advantages, we know that silver nanoparticles can be extremely dangerous to the body’s organs. Therefore, to overcome this problem researchers and scientists focus their attention on the incorporation of silver nanoparticles in a silica matrix. These structures, called core-shell, exhibit improved properties in relation to the same metallic nanoparticles, such as controlled size and additives that prevent agglomeration.

Thus, the primary goal of the present study was to prepare and characterize new types of core-shell nanomaterials for potential use as the components of cosmetic or medical products applied onto the skin. Insights from studies of the proposed nanostructures will contribute to the selection of those with the lowest cytotoxicity and the highest hydrophilicity.

### 3.1. Synthesis and Characterization of Nanocomposite

**Scheme 1:**
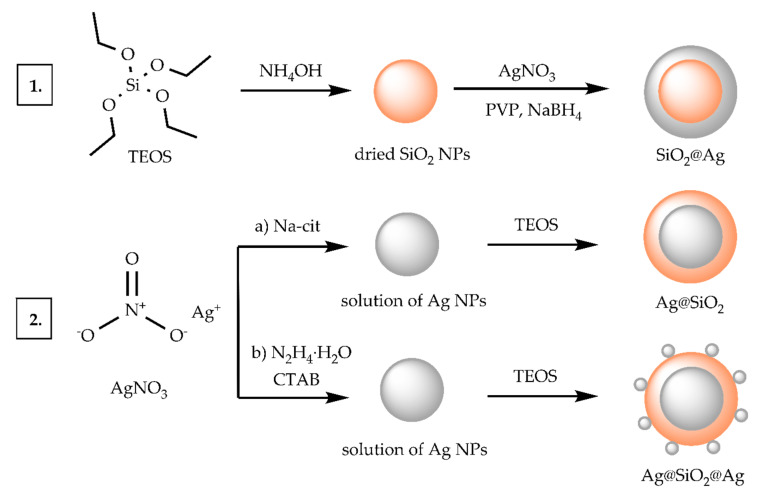
The synthesis routes of the presented nanostructures based on silver and silica. The numbers in the diagram correspond to the synthesis of the respective structure—(**1**) SiO_2_@Ag, (**2**) (a) Ag@SiO_2_, (b) Ag@SiO_2_@Ag.

As illustrated in Scheme 1, the preparation of different types of core-shell nanostructures was achieved in two steps. In the case of SiO_2_@Ag nanostructures, the preparation started from the synthesis of silica nanoparticles (SiO_2_ NPs). We used the Ströber method, which involves the hydrolysis and then condensation of tetraethoxysilane (TEOS). This reaction is possible due to the basic pH obtained by the addition of ammonia, water, and an alcoholic solvent—in this case, ethanol. Under these conditions, the hydrolysis takes place, leading to the attachment of hydroxyl groups. Next, SiO_2_ nanosparticles were centrifuged, washed with ethanol, and dried. In the second step, Ag NPs were formed from the silver nitrate in the presence of NaBH_4_ as a reductant. In addition, polyvinylpyrrolidone (PVP) was added to prevent agglomeration, and ammonia was added to form Ag(NH_3_)^2+^ ions. Finally, due to the electrostatic attraction of Si-O^−^ anions formed on the surface of SiO_2_ NPs and the Ag(NH_3_)^2+^ cation, the core-shell nanostructures were obtained (see Scheme 1-a). In the case of Ag@SiO_2_ preparation and sandwich nanostructures of Ag@SiO_2_@Ag type, the synthesis route was started from the reduction in silver nitrate in an aqueous solution. Sodium citrate (Na-cit) was used as a reducing agent for the synthesis of Ag@SiO_2_ nanostructures (Scheme 1-a). However, in the case of the Ag@SiO_2_@Ag nanostructures preparation, hydrazine was used as a reductant (Scheme 1-b). Additionally, in the case of sandwich nanomaterials, we added cetrimonium bromide (CTAB) as a surfactant to prevent the surface aggregation of Ag NPs. In the next step, the silver nanoparticles were coated with silica using the previously mentioned Ströber method.

### 3.2. Transmission Electron Microscopy—Morphology Analysis

**Figure 1:**
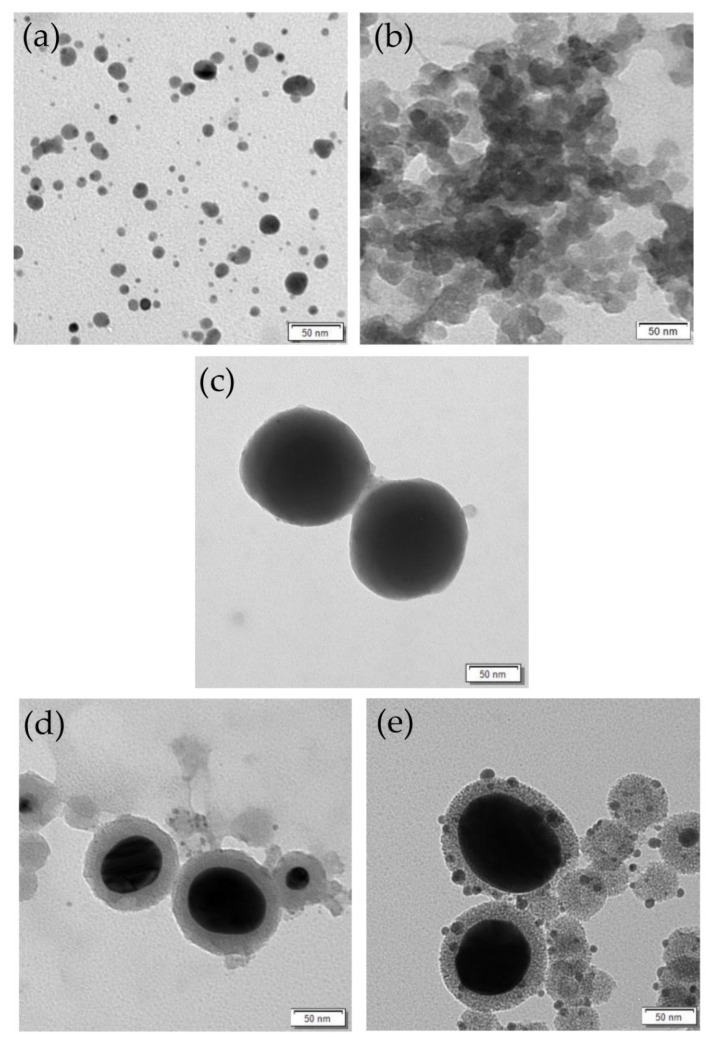
TEM photo of (**a**) Ag NPs, (**b**) SiO_2_ NPs, (**c**) SiO_2_@Ag, (**d**) Ag@SiO_2_, and (**e**) Ag@SiO_2_@Ag.

The morphology of the obtained structures was determined using transmission electron microscopy (TEM), and all nanostructures were spherical in shape (Figure 1). Figure 1a shows silver nanoparticles (Ag NPs) with a diameter between 5 and 30 nm. The SiO_2_ NPs are presented in Figure 1b, where their high agglomeration is visible, and the diameter of a single particle is about 30 nm.

Figure 1c–e shows a typical micrograph of the studied core-shell nanocomposite. The bright area depicts silica, and the dark area depicts nanosilver. As for SiO_2_@Ag (Figure 1c), it is difficult to observe the size of the individual components of the material, but the diameter of the entire structure can be determined, which is about 100 nm. Moreover, in the case of SiO_2_@Ag, we observed that the silver nanoparticles agglomerating on the surface form a uniform coating. Figure 1d shows the Ag@SiO_2_ core-shell, where the silver core is 20 to 80 nm in diameter, while the shell measures about 20 nm. In the case of the Ag@SiO_2_@Ag structure (Figure 1e), starting from the center of the structure, the diameter of the metallic core is 70–90 nm, the silica shell is 15 nm, and the nanoparticles attached to them are of small size, 10–15 nm.

### 3.3. UV-Vis Spectroscopy

The UV-Vis spectroscopy method enables observation of the maximum absorption of silver nanoparticles that is in the range of 350–450 nm. In the case of silica in the 300–700 nm measurement range, no maximum absorption was observed [48].

**Figure 2:**
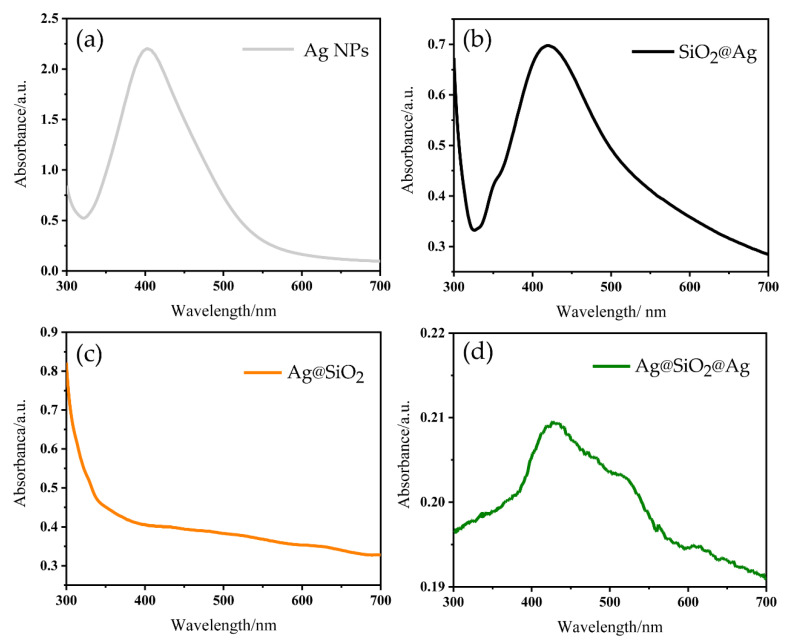
Absorption spectra of (**a**) Ag NPs, (**b**) SiO_2_@Ag, (**c**) Ag@SiO_2_, and (**d**) Ag@SiO_2_@Ag.

The spectra of Ag NPs, SiO_2_@Ag, Ag@SiO_2,_ and Ag@SiO_2_@Ag are presented in Figure 2. Ag NPs were prepared in a solution, and we observed here a characteristic absorption maximum at about 400 nm. However, in the case of core-shell nanostructures, the spectra were measured as a suspension in water. For SiO_2_@Ag, a maximum was observed at 420 nm, which corresponds to a coating of Ag NPs. The Ag@SiO_2_ nanostructures were characterized by the lack of maximum absorption, which is probably due to the high thickness of the SiO_2_ coating. In the case of Ag@SiO_2_@Ag structures, a low-intensity peak at 430 nm was noticeable, which is also related to the presence of Ag NPs on the surface.

### 3.4. FT-IR Spectroscopy Analysis

**Figure 3:**
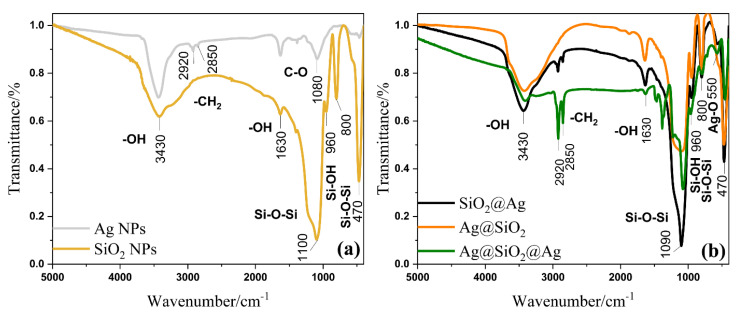
Fourier-transform infrared spectra for (**a**) Ag NPs, SiO_2_ NPs and (**b**) SiO_2_@Ag, Ag@SiO_2_, Ag@SiO_2_@Ag.

Additionally, we carried out FT-IR measurements to gain a deeper insight into the chemical composition of the obtained materials. Previous studies show that the FT-IR technique enables the detection of various groups on the surface of the obtained materials. Figure 3 shows the FT-IR spectra of Ag NPs, SiO_2_ NPs, SiO_2_@Ag, Ag@SiO_2_, and Ag@SiO_2_@Ag. The spectrum of Ag NPs includes several bands that can come from ethanol. The peak located at 3430 cm^−1^ is associated with the -OH stretching vibrations that come from alcohol. Two bands located at 2920 and 2850 cm^−1^ correspond to C-H stretching vibrations, both characteristic of ethanol. The weak band located at 1630 cm^−1^ can be associated with the bending vibration of the hydrogen in the -OH group. In addition, the peak placed at 1080 cm^−1^ is associated with the C-O stretching vibrations from ethanol [49].

The FT-IR spectrum of SiO_2_ NPs contains several bands characteristic of silica. The broad band in the 2900–3500 cm^−1^ with maximum placed at 3430 cm^−1^ and weak peak located at 1630 cm^−1^ can be assigned to -OH stretching and bending vibration, respectively, due to the presence of water molecules adsorbed onto the silica surface. The set of the bands located at 1100, 800, and 470 cm^−1^ corresponds to the asymmetric stretching vibrations, symmetric stretching vibrations, and bending vibration in Si-O-Si group, respectively. The characteristic band at 960 cm^−1^ can be assigned to silanol (Si-OH) groups [50,51].

Characteristic bands appeared in the FTIR spectrum recorded for SiO_2_@Ag nanostructure. The bands observed at 2920 and 2850 cm^−1^ can be assigned to the stretching vibrations in the -CH_2_ groups due to the polyvinylpyrrolidone (PVP) utilization during the synthesis [42]. Moreover, a low intensity peak at 550 cm^−1^ indicates the presence of Ag NPs on the SiO_2_ surface and belongs to Ag-O stretching vibration [52,53,54].

In the case of Ag@SiO_2_ nanostructures, the peaks indicating the presence of silica on the surface were presented above and are described in the details in our previous paper [42].

Furthermore, the FT-IR spectrum of Ag@SiO_2_@Ag sandwich nanostructure presents characteristic bands of this nanostructure. The bands placed at 2920 and 2850 cm^−1^ can be attributed to the C−H stretching vibration of methyl and methylene groups, which comes from CTAB [55]. In addition, the presence of silver in the outer shell exposes a peak placed at 550 cm^−1^, indicating the stretching vibration of Ag-O bond.

### 3.5. Thermogravimetric Analysis

**Figure 4:**
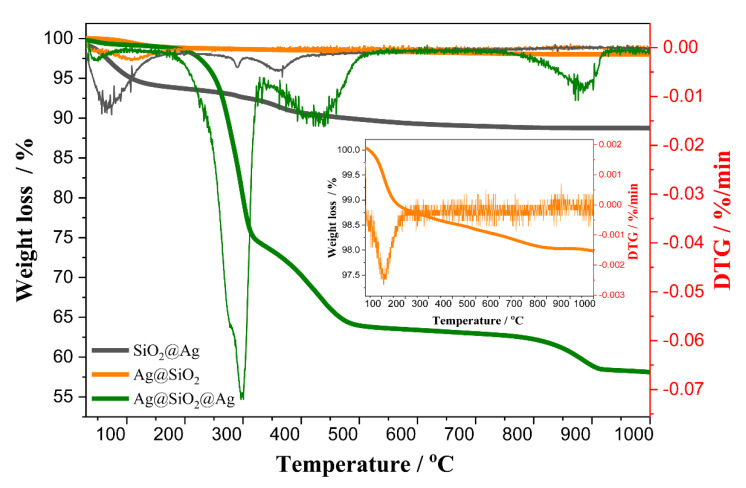
TG and DTG curves for SiO_2_@Ag, Ag@SiO_2_, and Ag@SiO_2_@Ag.

Thermogravimetric analysis (TGA) and derivative thermogravimetric analysis (DTG) for SiO_2_@Ag, Ag@SiO_2_, and Ag@SiO_2_@Ag are presented in Figure 4.

The decomposition of SiO_2_@Ag nanomaterial in air atmosphere occurs in two stages. The first weight loss is observed at the TG curve in the range of 65 and 290 °C. The weight loss of 5.32% is attributed to water evaporation, which is physically adsorbed on the material surface. In the temperature range between 290 and 360 °C, another significant weight loss of 3.48% is observed and could be assigned to the reduction in hydroxyl groups present on the surface of studied material. However, in the case of Ag@SiO_2_, a similar weight loss is observed, as in the previous papers [11,56,57].

The decomposition of Ag@SiO_2_@Ag nanomaterial occurs in three stages. The first small signal that is seen in DTG curve is also the result of water and alcohol evaporation. The weight loss of 25.11% observed in the region within 200 to 350 °C could be assigned to hydrolysis and polycondensation reactions of the unreacted alkoxy and hydroxy groups [58]. The second peak observed in DTG curves centered at 460 °C with the weight loss of 4.99% mainly corresponds to the composition of organic groups from TEOS used in the synthesis [27].

### 3.6. Contact Angle Measurement

A series of measurements of the contact angle was performed to determine the hydrophilicity of the tested nanoparticles, as well as the influence of the core-shell system type on these properties.

**Figure 5:**
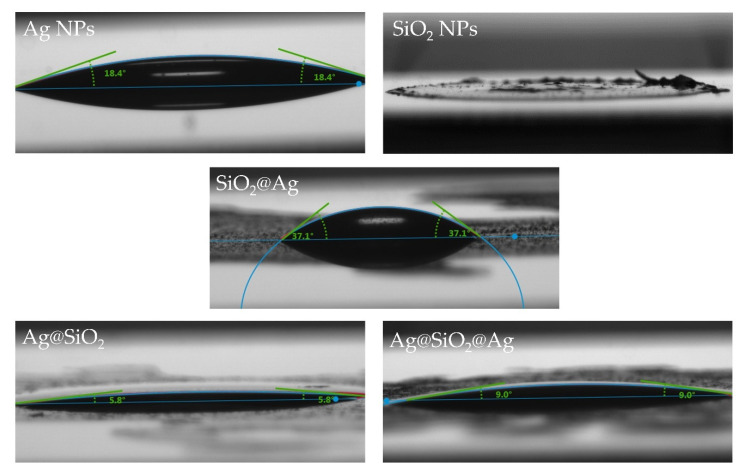
Photographs showing water contact angle measurement results for the measured samples Ag NPs, SiO_2_ NPs, SiO_2_@Ag, Ag@SiO_2_, and Ag@SiO_2_@Ag (measurements of Ag NPs and Ag@SiO_2_ reprinted (adapted) from [42] which is an open access article and permits unrestricted use)).

Generally, all the tested nanoparticles are hydrophilic and have a much lower contact angle value than the control sample (glass plate—55°) (Figure 5). Reference measurements were made for Ag NPs and SiO_2_ NPs, for which the contact angle was 18.4° and 0°, respectively. The contact angle of about 5° is a characteristic of the superhydrophilic class particles. Therefore, SiO_2_ NPs are superhydrophilic, while Ag@SiO_2_ are characterized by the highest hydrophilicity among all silver- and silica-based nanostructures. The present study shows that the presence of silver in the shell reduces the hydrophilicity of a nanoparticle. We observe this both for SiO_2_@Ag and for doping the SiO_2_ shell with silver for Ag@SiO_2_@Ag. Although wettability results from both the chemical nature and the roughness of the surface [42], in this case the investigated nanoparticles (Ag@SiO_2_ and SiO_2_@Ag) are similar in size; therefore, we may assume that the roughness is comparable. Here, we assume that the differences are only due to the chemical properties of these nanoparticles. Moreover, good wettability of Ag@SiO_2_ nanomaterial shows the promise of its utilization as a drug carrier in medicine and cosmetology.

### 3.7. Cytotoxic Effect of SiO_2_@Ag, Ag@SiO_2_, Ag@SiO_2_@Ag, and SiO_2_ Nanoparticles

The application of various nanostructures in cosmetic or medical products not only improves their sterility and stability but also overcomes the barrier of the cuticle. Thus, the nanostructures enable the cosmetic actives to enter the target site of the skin, perform the sustained release function, and solve various skin problems or cure diseases.

Silver nanoparticles are used as antibacterial materials in medical and cosmetic products due to their excellent antibacterial activity. Previous studies show that silver nanoparticles can destroy over 650 microorganisms compared with antibiotics. With the growing number of medical and cosmetic products containing nanosilver, its biological safety becomes an urgent issue. Silver nanoparticles possess particular physicochemical properties that determine the extent of their cytotoxicity in biological systems [59]. Unfortunately, recent studies show the cytotoxicity of silver nanoparticles on various types of cells, including human peripheral blood cells, rat liver cell, and mouse germ line cells [60]. Moreover, results of the studies indicate that silver nanoparticles exhibit intense toxicity upon some tissues and that after ingestion they can induce neurological problems, kidney damage, stomach upset, and skin irritation [61]. In [62], the authors provide comprehensive information about particle size and toxicity—that is, lower particle size is responsible for higher toxicity. Aggregation and sedimentation lead to a decrease in the activity of biologically active particles. Moreover, cytotoxicity not only depends on Ag NPs’ properties but also the organism’s variation plays a vital role.

However, to overcome the challenges that come along with the utilization of silver nanoparticles as a nanocarrier in medical or cosmetic products, we must consider their incorporation into a silica shell to limit cytotoxicity.

In line with the research trend concerning the cytotoxicity of silver nanoparticles, our recent studies showed that cytotoxicity depends on their physicochemical properties. According to our results, the large Ag NPs (100 nm) at low concentration of 0.6–5 ppm exhibited a higher cytotoxic effect on HaCaT and HDF cells, whereas the lower viability of HEM cells was observed at a concentration range of 2.5–5 ppm, compared with the smaller Ag NPs (20 nm). The determined IC_50_ parameter for the larger Ag NPs for HaCaT cells was 0.75 ppm and for HDF cells it was 0.92 ppm, while for the smaller Ag NPs for HaCaT cells it was >15 ppm, and for HDF it was 8.44 ppm [6].

**Figure 6:**
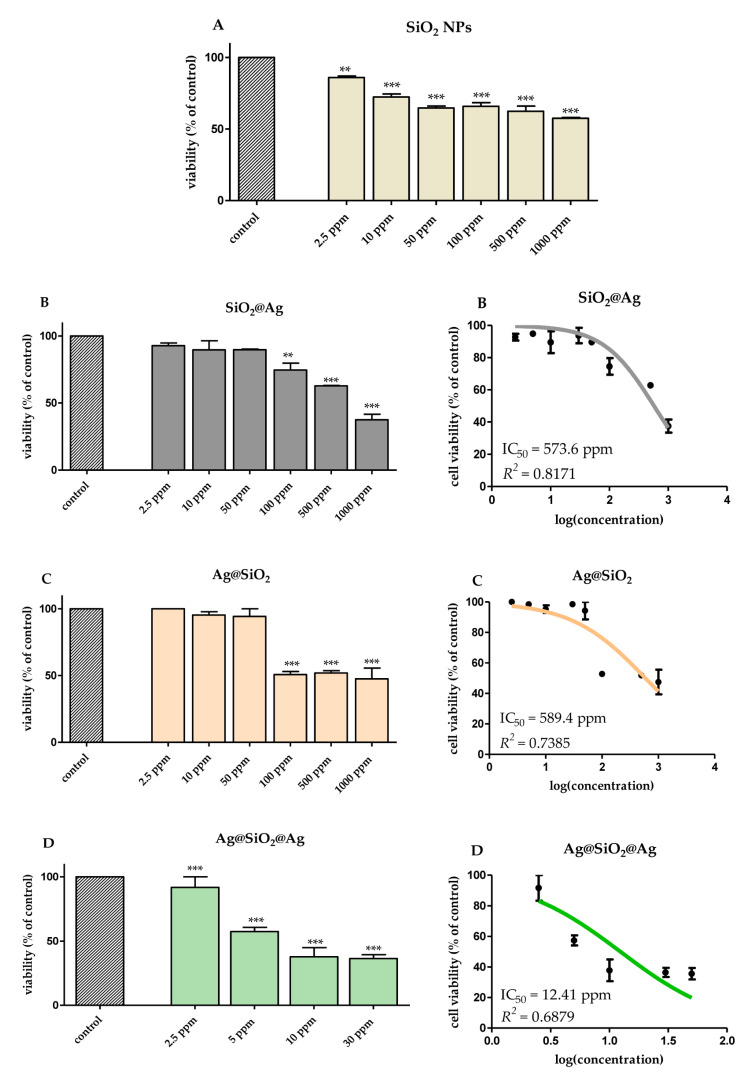
The viability of HaCaT keratinocytes cells after 24 h of exposure to (**A**) SiO_2_ NPs (diameter 30 nm), (**B**) SiO_2_@Ag, (**C**) Ag@SiO_2_, (**D**) Ag@SiO_2_@Ag evaluated using MTT test. Data are expressed as mean values ± SD from three separate experiments. ** *p* < 0.01; *** *p* < 0.001 versus control (left panel). The IC_50_ values were calculated using a nonlinear regression analysis for HaCaT cells exposed to (**B**) SiO_2_@Ag, (**C**) Ag@SiO_2_, (**D**) Ag@SiO_2_@Ag. Data are expressed as means ± SD for at least three replicates. *R*^2^—coefficient of determination (right panel).

**Figure 7:**
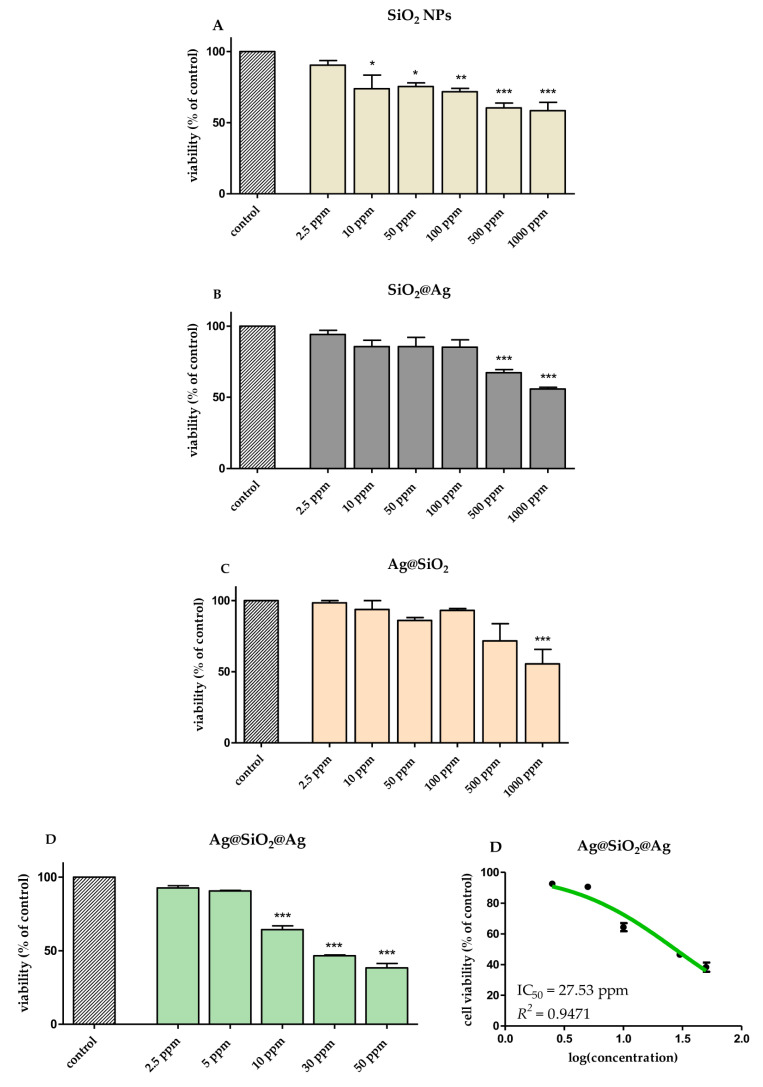
The viability of the HDF fibroblasts cells after 24 h of exposure to (**A**) SiO_2_ NPs (diameter 30 nm), (**B**) SiO_2_@Ag, (**C**) Ag@SiO_2_, (**D**) Ag@SiO_2_@Ag evaluated using MTT test. Data are expressed as mean values ± SD from three separate experiments. * *p* < 0.05 ** *p* < 0.01; *** *p* < 0.001 versus control (left panel). The IC_50_ values were calculated using a nonlinear regression analysis for HDF cells exposed to (**D**) Ag@SiO_2_@Ag. Data are expressed as means ± SD for at least three replicates. *R*^2^—coefficient of determination (right panel).

These studies cover silica and silver-based nanostructures—namely, SiO_2_@Ag, Ag@SiO_2_, Ag@SiO_2_@Ag, and SiO_2_. We evaluated the influence of various nanostructure types on the viability of immortalized human keratinocytes—HaCaT (Figure 6) and human dermal fibroblasts—HDF (Figure 7) using MTT assay in the 1 to 1000 ppm range. The obtained results show that Ag@SiO_2_@Ag significantly decreased the cell viability at low concentration in both tested cell lines (IC_50_ = 12.41 ppm for HaCaT, IC_50_ = 27.53 ppm for HDF). It is supposed that the cytotoxicity of Ag@SiO_2_@Ag is caused by the small size of Ag NPs on the surface, i.e., 10–15 nm. SiO_2_@Ag and Ag@SiO_2_ nanostructures exhibited minimal toxic effect on cells and were characterized by very high half-maximal inhibitory concentration (Table 1 and Table 2). Unexpectedly, the low cytotoxicity of SiO_2_@Ag is probably due to their larger diameter the higher proportion of silica core to silver shell and agglomeration of silver nanoparticles on the silica surface. What is important is that the silica used in the tested nanostructures did not cause any toxicity at used concentrations (IC_50_ > 1000 ppm). The obtained results suggest that the silver nanoparticles modified by silica have dramatically decreased toxicity. In addition, Yan Li et al. [63] showed that genotoxicity was related to the size and coating materials of AgNPs. 

## 4. Conclusions

We successfully developed the preparation method of the structurally stable materials based on silver and silica nanoparticles, including SiO_2_@Ag, Ag@SiO_2_, and sandwich structures—Ag@SiO_2_@Ag. We confirmed the morphology of these structures using methods such as transmission electron microscopy (TEM), UV-Vis spectroscopy, and FT-IR spectroscopy. Measurements of the contact angle showed that Ag@SiO_2_ is a superhydrophilic nanomaterial, which influences its potentially wide application.

The obtained results suggest that modification of silver nanoparticles by silica dramatically decreases the toxicity of silver. Moreover, Ag@SiO_2_ showed the lowest cytotoxicity against the two skin cell lines used in the study—keratinocytes (HaCaT) and fibroblasts (HDF).

Therefore, the designed Ag@SiO_2_ nanostructures are promising candidates for a potential use as a new nanocarrier drug-delivery system in the fields of medicine and cosmetology.

## Figures and Tables

**Table 1:** IC_50_ and logIC_50_ for HaCaT of Ag NPs (20 nm), Ag NPs (100 nm) reported in [6], and SiO_2_, SiO_2_@Ag, Ag@SiO_2_, and Ag@SiO_2_@Ag for HaCaT cells.

	HaCaT					
	Ag NPs (20 nm)	Ag NPs (100 nm)	SiO_2_ NPs	SiO_2_@Ag	Ag@SiO_2_	Ag@SiO_2_@Ag
**IC_50_**	>15 ppm	0.75 ppm	>1000 ppm	573.6 ppm	589.4 ppm	12.41 ppm
**logIC_50_**	-	−6.135 ± 0.071	-	2.759 ± 0.063	2.770 ± 0.132	1.094 ± 0.097

**Table 2:** IC_50_ and log IC_50_ of Ag NPs (20 nm), Ag NPs (100 nm) reported in [6], and SiO_2_ NPs, SiO_2_@Ag, Ag@SiO_2_, and Ag@SiO_2_@Ag for HDF cells.

	HDF					
	Ag NPs (20 nm)	Ag NPs (100 nm)	SiO_2_ NPs	SiO_2_@Ag	Ag@SiO_2_	Ag@SiO_2_@Ag
**IC_50_**	8.44 ppm	0.92 ppm	>1000 ppm	>1000 ppm	>1000 ppm	27.53 ppm
**logIC_50_**	−5.074 ± 0.1040	−6.04 ± 0.052	-	-	-	1.044 ± 0.0418

## Data Availability

The data presented in this study are available on request from the corresponding author.
